# Improved Performance of ELISA and Immunochromatographic Tests Using a New Chimeric A2-Based Protein for Human Visceral Leishmaniasis Diagnosis

**DOI:** 10.1155/2021/5568077

**Published:** 2021-04-28

**Authors:** Maria Marta Figueiredo, Anna R. R. dos Santos, Lara C. Godoi, Natália S. de Castro, Bruno C. de Andrade, Sarah A. R. Sergio, Selma M. B. Jerônimo, Edward J. de Oliveira, Ruth T. Valencia-Portillo, Lucilândia M. Bezerra, Hiro Goto, Maria C. A. Sanchez, Caroline Junqueira, Santuza M. R. Teixeira, Flávio G. da Fonseca, Ricardo T. Gazzinelli, Ana Paula Fernandes

**Affiliations:** ^1^Centro de Tecnologia em Vacinas da Universidade Federal de Minas Gerais, Belo Horizonte, Minas Gerais, Brazil; ^2^Universidade Estadual de Minas Gerais, Divinópolis, Minas Gerais, Brazil; ^3^Detechta Biotecnologia S.A, Brazil; ^4^Colégio Técnico da Universidade Federal de Minas Gerais, Belo Horizonte, Minas Gerais, Brazil; ^5^Departamento de Bioquímica, Universidade Federal do Rio Grande do Norte, Natal, Brazil; ^6^Instituto René Rachou, Fundação Oswaldo Cruz, Belo Horizonte, Minas Gerais, Brazil; ^7^Instituto de Medicina Tropical da Faculdade de Medicina, Universidade de São Paulo, São Paulo, São Paulo, Brazil; ^8^Departamento de Pós-Graduação em Ciência Animal, Universidade Federal de Goiás, Goiânia, Goiás, Brazil; ^9^Departamento de Medicina Preventiva, Faculdade de Medicina, Universidade de São Paulo, São Paulo, São Paulo, Brazil; ^10^Departamento de Bioquímica e Imunologia, Universidade Federal de Minas Gerais, Belo Horizonte, Minas Gerais, Brazil; ^11^Departamento de Microbiologia, Instituto de Ciências Biológicas (ICB/UFMG), Belo Horizonte, Minas Gerais, Brazil; ^12^Departamento de Análises Clínicas e Toxicológicas, Faculdade de Farmácia, Universidade Federal de Minas Gerais, Belo Horizonte, Minas Gerais, Brazil

## Abstract

**Methods:**

A total of 1028 sera samples were used for the development and validation of ELISA (321 samples from *L. infantum*-infected patients, 62 samples from VL/AIDS coinfected patients, 236 samples from patients infected with other diseases, and 409 samples from healthy donors). A total of 520 sera samples were used to develop and validate ICT (249 samples from *L. infantum*-infected patients, 46 samples from VL/AIDS coinfected patients, 40 samples from patients infected with other diseases, and 185 samples from healthy donors). *Findings*. Using the validation sera panels, DTL-4-based ELISA displayed an overall sensitivity of 94.61% (95% CI: 89.94-97.28), a specificity of 99.41% (95% CI: 96.39-99.99), and an accuracy of 97.02% (95% CI: 94.61-98.38), while for ICT, sensitivity, specificity, and accuracy values corresponded to 91.98% (95% CI: 86.65-95.39), 100.00% (95% CI: 96.30-100.00), and 95.14% (95% CI: 91.62-97.15), respectively. When testing sera samples from VL/AIDS coinfected patients, DTL-4-ELISA displayed a sensitivity of 77.42% (95% CI: 65.48-86.16), a specificity of 99.41% (95% CI: 96.39-99.99), and an accuracy of 93.51% (95% CI: 89.49%-96.10%), while for DTL-4-ICT, sensitivity was 73.91% (95% CI: 59.74-84.40), specificity was 90.63% (95% CI: 81.02-95.63), and accuracy was 82.00% (95% CI: 73.63-90.91).

**Conclusion:**

DTL-4 is a promising candidate antigen for serodiagnosis of VL patients, including those with VL/AIDS coinfection, when incorporated into ELISA or ICT test formats.

## 1. Introduction

Human visceral leishmaniasis (VL) is one of the world's most neglected diseases, largely affecting low-socioeconomic-level individuals, mainly in developing countries [1, 2, 3]. When not treated, VL is fatal in 90%-100% of cases [4, 5]. According to the World Health Organization (WHO) estimates, 50,000 to 90,000 new VL cases occur each year worldwide [6]. The zoonotic VL caused by *Leishmania* (*Leishmania*) *infantum* occurs in Mediterranean countries (North Africa and Europe); Southeast Europe; Middle East; Central Asia; and North, Central, and South America (Mexico, Venezuela, Brazil, and Bolivia) [7]. The zoonotic VL transmission areas have expanded lately due to the migration of people from rural to urban areas [8]. In 2018, more than 95% of new VL cases occurred in Brazil and nine countries of Asia and Africa [6], and 90% of VL cases in America occurred in Brazil [4].

Progress towards noninvasive, easy-to-perform, and highly accurate diagnosis of leishmaniasis depends on discovering suitable biomarkers and their use in sensitive, specific, and amenable diagnostic platforms to both laboratory and field conditions. Anti-*Leishmania* antibodies' detection is an important diagnostic alternative and may be achieved using several different test platforms. The most used serological methods are enzyme-linked immunosorbent assay (ELISA) and immunochromatographic tests [9–12]. Nevertheless, on serological assays, the diagnosis of cases with low or undetectable anti-*Leishmania* antibodies is a drawback and causes a drop in the test sensitivity. Another limitation of serological tests is cross-reactivity with other pathogens, which decreases the specificity. The low positive and negative predictive values resulting from these deficiencies generate uncertainties in the diagnostic accuracy [13].

To overcome the limitations in serological diagnosis, several groups have proposed the use of recombinant antigens comprising mapped and repetitive epitopes that can improve specificity and sensitivity of antibody detection [9, 11, 14–18]. In agreement, incorporating the recombinant rK39 or K28 antigens on immunochromatography platforms for VL serological diagnosis (rapid tests) represented a significant improvement since besides being faster and easier to perform, rapid tests with recombinant antigens are more accurate compared with assays based on total antigens [19, 20].

The recombinant protein A2 has also emerged as a promising antigen for VL serodiagnosis, which could be, in part, attributed to the repetitive structure of B cell epitopes present in A2 [9, 21–24]. Based on these previous studies, we designed a new chimeric protein containing part of the repetitive epitopes from A2 and other repetitive proteins expressed only by the visceralizing species of *Leishmania*, named DTL-4. Here, we report this chimeric recombinant antigen's performance in ELISA and immunochromatographic tests, using samples of patients from Brazilian endemic areas presenting confirmed clinical and laboratory diagnosis of VL. The results show that DTL-4, regardless of the diagnostic platform used, is a reliable and efficient antigen capable of detecting human VL cases with improved accuracy.

## 2. Methods

### 2.1. Study Design

The performance of the DTL-4 chimeric protein was evaluated for the development of kits for serological diagnosis, ELISA, and immunochromatographic tests (ICTs). According to good clinical practice principles, the study was approved by local authorities and was carried out between 2016 and 2019.

### 2.2. Ethics Statement

This study complied with Ethical Principles in Human Research and resolutions CNS 466 of 12/12/2012 and CNS 441 of 05/12/2011 and was approved by the Research Ethics Committee/UFMG (CAAE: 67820516.8.1001.5149). The samples collected in Aracaju (Sergipe State), Bauru (São Paulo State), Campo Grande (Mato Grosso do Sul State), and Natal (Rio Grande do Norte State) are part of Research Protocol no. 490/11. The samples from Palmas (Tocantins State) and Belo Horizonte (Minas Gerais State) were approved for this project (CAAE: 67820516.8.1001.5149). The samples from São Paulo (São Paulo State) are part of Protocol no. 529.638 of 01/22/2014. Samples from patients with HIV/*Leishmania* coinfection, collected in Campo Grande (Mato Grosso do Sul State) and Rio de Janeiro (Rio de Janeiro State), are part of Research Protocol no. 1021/09. Samples from patients with other infectious diseases are part of Research Protocol no. 339/08.

### 2.3. Patients and Healthy Donors

**Figure 1:**
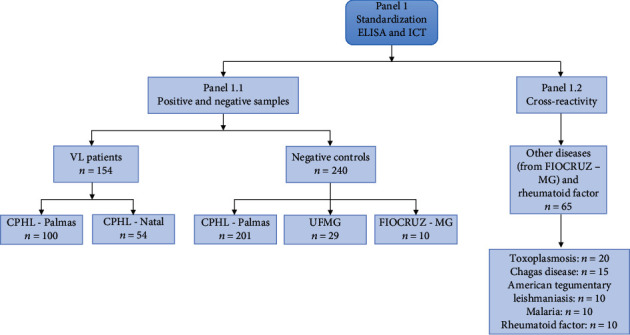
Sera samples used to standardize ELISA and ICT and the origin of the samples (panel 1.1 and panel 1.2). *n*: number of samples; VL: visceral leishmaniasis; CPHL-Palmas: Central Public Health Laboratory of Palmas (Tocantins State); CPHL-Natal: Central Public Health Laboratory of Natal (Rio Grande do Norte State); UFMG: Universidade Federal de Minas Gerais of Belo Horizonte (Minas Gerais State); FIOCRUZ, MG: René Rachou Institute (FIOCRUZ, Minas Gerais State).

The healthy donors included in the study did not display any symptoms suggestive of leishmaniasis when the blood samples were collected. Also, they showed no suggestive signs of any other infectious disease. VL patients consisted of women and men over 18 years old. All *L. infantum*-infected patients presented clinical symptoms of VL. Controls with possible cross-reactivity consisted of patients with a confirmed diagnosis of other diseases (Figure 1). Patients coinfected with *Leishmania*/HIV were also studied. These samples were obtained at several centers and were characterized at each center using routine diagnostic methods.

Initially, to develop ELISA and ICT using DTL-4, sera samples (Figure 1) were obtained at the Central Public Health Laboratory (CPHL) in Palmas (Tocantins State), CPHL in Natal (Rio Grande do Norte State), and UFMG in Belo Horizonte (Center for Vaccine Technology (CT-Vacinas) from Universidade Federal de Minas Gerais (Belo Horizonte, Minas Gerais State) and Fundação Oswaldo Cruz (Centro de Pesquisas René Rachou, Belo Horizonte, Minas Gerais State)). A total of 154 samples of patients with a confirmed VL diagnosis by either direct parasite microscopic or molecular detection, whose characteristics are described in the flowchart presented in Figure 1, were employed. Samples from patients from CPHL-Palmas were submitted to an indirect immunofluorescence (IFI) assay with *Leishmania* promastigotes, and samples from CPHL-Natal were assayed with both a house soluble leishmania promastigote antigen and rK39-based ELISA (Figure 1, panel 1.1). Negative sera samples from 240 healthy controls, with no symptoms of VL, also composed panel 1. To investigate cross-reactivity, 55 sera samples from subjects with a previously confirmed diagnosis of other parasitic diseases (toxoplasmosis, *n* = 20; Chagas' Disease, *n* = 15; American tegumentary leishmaniasis, *n* = 10, and malaria, *n* = 10) and rheumatoid factor (*n* = 10) were used (Figure 1, panel 1.2).

All positive and negative samples were further tested by in-house ELISA with a total extract of *L. infantum* (ELISA_EXT_) performed at René Rachou Institute (FIOCRUZ, Minas Gerais), and with ELISA recombinant protein (rK39 Rekom Biotech®) provided by BioClin Quibasa Ltda. (Belo Horizonte, Minas Gerais), and the commercial immunochromatographic test IT-LEISH® (Bio-Rad Laboratories, Inc.), performed at the CT-Vacinas.

**Figure 2:**
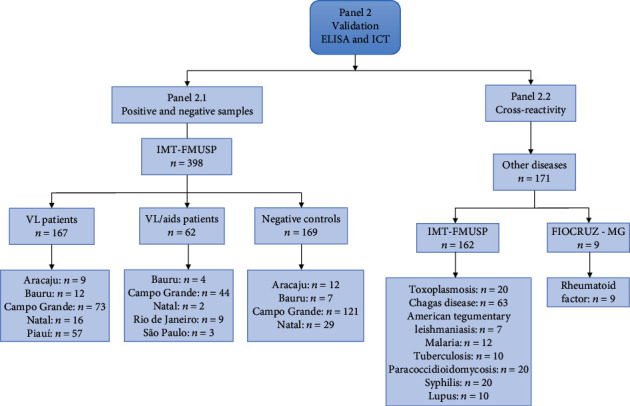
Sera samples used to validate the ELISA and ICT kits and the origin of the samples (panel 2.1 and panel 2.2). *n*: number of samples; VL: visceral leishmaniasis; VL/AIDS: coinfected patients; FIOCRUZ-MG: René Rachou Institute (FIOCRUZ, Minas Gerais State); IMT-FMUSP: Instituto de Medicina Tropical da Faculdade de Medicina, Universidade de São Paulo (São Paulo State). Aracaju, Bauru, Campo Grande, Natal, Rio de Janeiro, São Paulo are Brazilian cities; Piauí is a Brazilian state.

After the establishment of the conditions for the best performance of DTL-4-ELISA and ICT, the tests were submitted to external validation at the Instituto de Medicina Tropical da Faculdade de Medicina, Universidade de São Paulo (IMT-FMUSP), São Paulo State, Brazil, with another sample panel (Figure 2, panel 2) with 569 samples. Panel 2.1 included sera from 167 *L. infantum*-infected patients from distinct localities in Brazil (Aracaju, Sergipe State; Piauí State; Bauru, São Paulo State; Campo Grande, Mato Grosso do Sul State; and Natal, Rio Grande do Norte State), and sera of 169 healthy individuals, living in an endemic area and negative by DAT (Aracaju, Sergipe State; Bauru, São Paulo State; Campo Grande, Mato Grosso do Sul State; Natal, Rio Grande do Norte State). The levels of DTL-4 antibodies were also assessed in a panel of sera from 62 patients previously diagnosed with VL/AIDS coinfection (Bauru, São Paulo State; Campo Grande, Mato Grosso do Sul State; Natal, Rio Grande do Norte State; Rio de Janeiro, Rio de Janeiro State; and São Paulo, São Paulo State). The *L. infantum* infection was diagnosed using the microscopic examination of the lymph node or bone marrow aspirate for *Leishmania* detection [25] and by direct agglutination test (DAT) [26]. The confirmation of HIV infection was made according to the Ministry of Health of Brazil [27].

To assess the interference of antibodies against other diseases with DTL-4-ELISA and ICT, 167 positive samples (Figure 2, panel 2.1) and 311 negative samples for VL (140 samples from panel 2.1 and 171 samples from panel 2.2—see Figure 2) were submitted to the interference test. Panel 2.2 was composed of seven positive samples for tegumentary leishmaniasis, 63 samples positive for Chagas disease (*Trypanosoma cruzi*), 12 positive samples for malaria (*Plasmodium vivax*/*Plasmodium falciparum*), 20 samples positive for toxoplasmosis (*Toxoplasma gondii*), 10 samples positive for tuberculosis, 20 samples positive for syphilis, ten samples positive for lupus erythematosus, 20 samples positive for paracoccidioidomycosis, and nine samples with increased levels of rheumatoid factor.

### 2.4. Cloning, Expression, and Purification of Recombinant DTL-4 Protein

The *DTL-4* gene synthesized and cloned into the *E. coli* expression vector pET24a was purchased from GenScript (Piscataway, NJ, EUA). *E. coli* BL21 (DE3) cells transformed with pET24a/*DTL-4* were grown at 37°C in 2 L of Luria-Bertani's medium (LB) with 50 *μ*g/mL kanamycin until the optical density of 600 nm reached 0.6 before adding isopropyl beta-D-thiogalactopyranoside (IPTG). After 3 hours of incubation, the cells were harvested by centrifugation, and the bacterial cell pellet was resuspended in a solution containing 20 mM Tris-HCl (pH 8), 100 mM NaCl, 5 mM dithiothreitol (DTT), 5 mM benzamidine, and 1 mM phenylmethylsulfonyl fluoride (PMSF) and lysed in a homogenizer. Aliquots of total cell extracts were collected and analyzed by 12% sodium dodecyl sulfate-polyacrylamide gel electrophoresis (12% SDS-PAGE). The his-tagged protein was purified using the AKTA Prime Plus System (GE Healthcare). The suspension was loaded onto a Ni^2+^-charged chelating Sepharose HisTrap HP (GE Healthcare). Contaminants were washed away with a solution containing 20 mM Tris-HCl (pH 8) and 100 mM NaCl. The recombinant protein was then eluted with a solution containing 20 mM Tris-HCl (pH 8), 100 mM NaCl, and increasing amounts of imidazole, starting from 0 to 500 mM. Purified protein samples were analyzed by 12% SDS-PAGE. The protein was extensively dialyzed against 20 mM Tris-HCl (pH 8), 100 mM NaCl, and 10% glycerol at 4°C and frozen. Further detailing of the protein and its amino acid sequence will not be revealed due to pending patent issues.  Table 1 describes some characteristics of the recombinant DTL-4 protein.

The A2 recombinant protein (rA2) was produced as previously described [28]. The rK39 protein was purchased at Rekom Biotech® (Spain) and kindly provided by BioClin Quibasa Ltda. (Brazil).

### 2.5. DTL-4-ELISA

The presence of anti-DTL-4 IgG antibodies in plasma and serum was evaluated by the indirect ELISA method (enzyme-linked immunosorbent assay), using the purified recombinant DTL-4 antigen. ELISA plates (Costar®) were coated with 0.2 *μ*g/well of recombinant protein diluted in carbonate-bicarbonate buffer (pH 9.6) (100 *μ*L/well). The plates were incubated at 4°C for 18 hours and blocked with 1% BSA (280 *μ*L/well) at 25°C for 2 hours. Plasma or serum samples were added to each well at a final dilution of 1 : 100. The antibody-antigen binding was detected by the addition of peroxidase-conjugated goat anti-human IgG FAPON® (1 : 100,000). The presence of bound IgG was detected using tetramethylbenzidine (MOSS), and the reaction stopped by the addition of 0.5 M H_2_SO_4_. The optical density (OD) was measured at 450 nm using a Multiskan GO microplate spectrophotometer (Thermo Fisher Scientific). The results were expressed as optical density (OD). The cut-off points were set at three standard deviations above the mean optical density read at 450 nm. RI values > 1.1 were considered positive [9].

Initially, a titration was performed to determine the best amount of protein and serum dilution to be applied in individual serum evaluations by ELISA, using pools of samples with ten VL-positive sera and ten negative sera. We also compared the antibody levels against rA2, rK39, and rDTL-4 with the same sample pools, using in-house ELISA, since DTL-4 contains A2 epitopes, while rK39 is widely used as a recombinant antigen in several commercial tests. Next, we evaluated DTL-4-ELISA using 459 samples (positive and negatives samples from Figure 1, panel 1).

### 2.6. Repeatability, Reproducibility, Homogeneity, and Stability of DTL-4-ELISA

The DTL-4-ELISA intra-assay repeatability was determined using one positive and one negative sample in eight determinations to establish the coefficient of variation (% CV is equal to the standard deviation divided by the mean multiplied by 100) among measurements, within the same plate. A second test was performed to evaluate the test reproducibility, determining the interassay CV. To determine that, three samples were evaluated in three consecutive days, in eight measurements. A third test was carried out to evaluate the solid phase's homogeneity, applying the same samples in different plate wells. A fourth test was carried out to assess the reagents' stability in the solid phase, and each test solution, separately, at 37°C [29], to predict the shelf life of the kits stored under appropriate conditions. The cut-off was determined using one hundred samples from healthy donors from panel 1.1, and the cut-off points were defined as the mean plus three standard deviations.

### 2.7. Immunochromatographic Test (ICT)

**Figure 3:**
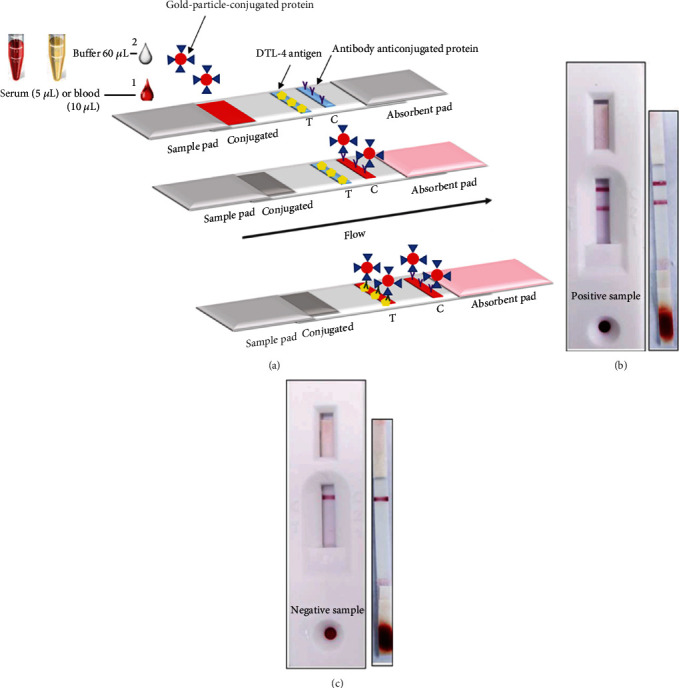
Representation of the ICT to detect IgG anti-*Leishmania* antibody. (a) The rapid test scheme developed for the detection of IgG anti-*L. infantum*. (b) Positive sample and (c) negative sample.

A lateral flow-based doubled antigen immunochromatographic test (ICT) for antibody detection was assembled using the DTL-4 protein. The purified DTL-4 antigen was dispensed as the test line (T), and the *Staphylococcus aureus* protein A was dispensed as the control line (C), both in the detection zone of the nitrocellulose membrane. Protein A labeled with a signal generator colloidal gold was used to detect the samples' antibodies as a control test line. For conjugation, either the DTL-4 protein or protein A were mixed with colloidal gold (Sigma-Aldrich) and incubated at room temperature. To detect specific antibodies, we immobilized the DTL-4 protein onto the nitrocellulose membrane. The conjugate was adsorbed to the glass fiber and dried in a low humidity room. When in contact with sera, plasma, or peripheral blood containing anti-*L. infantum* antibodies, these first reacted with the colloidal gold conjugates on the conjugation pad. As the colloidal gold complex flows through the capture site, antibodies reacted with the antigens at the test line site, leading to the formation of a visible colored line. In the absence of specific antibodies (negative samples), no reactivity is observed at this site (Figure 3). The test was considered valid only if the control line could be clearly seen. Several tests were carried out to define the membranes' best control and test lines' location, type of plastic support, size of gold particles, sample volume, and buffer for lateral flow.

The sensitivity, specificity, and accuracy of the developed ICT test were evaluated against 520 samples, composed of 185 samples from healthy donors, from panels 1 and 2 (Figure 1 and 2) and 249 samples from *L. infantum*-infected patients from the Central Public Health Laboratory of Palmas (87 samples from panel 1.1—see Figure 1) and Instituto de Medicina Tropical of São Paulo (162 samples from panel 2.1—see Figure 2), and 46 samples from VL / AIDS coinfected patients (panel 2.1—see Figure 2). Cross-reactivity was evaluated with ten samples of each disease (Chagas disease, American tegumentary leishmaniasis, and malaria) and of rheumatoid factor (40 samples from panel 1.2—see Figure 1). The strip stability after the unpacking was assessed by holding the cassettes at room temperature for two hours and testing them with positive and negative samples. The accelerated aging stability was assessed, keeping the strips at 37°C for 7, 14, 21, and 28 days and testing them on days 0, 7, 14, 21, and 28 days before the test.

### 2.8. Statistical Analysis

The results were analyzed using GraphPad Prism (version 7.0 for Windows). The lower limits of positivity (cut-off) for DTL-4-ELISA were established for optimal sensitivity, specificity, and accuracy using ROC (receiver operating characteristic) analysis. The D'Agostino-Pearson normality test, the Shapiro-Wilk normality test, and the Kolmogorov-Smirnov normality test were used to determine whether a variable was normally distributed. The Mann–Whitney test and ANOVA were also used, and significant differences were considered when *p* < 0.05. The diagnostic capacity of DTL-4 was measured by assessing its sensitivity, specificity, and accuracy with 95% confidence intervals (CI). Kappa index was calculated according to Cohen [30] and interpreted according to Landis and Koch [31] to assess the agreement between ELISA and ICT with the serological reference methods: 1.00–0.81 = excellent; 0.80–0.61 = good; 0.60–0.41 = moderate; 0.40–0.21 = weak; and 0.20–0.00 = negligible agreement. McNemar's test was used to estimate the statistical differences between ELISA and ICT. The significance of the difference between proportions was tested using the chi-square with Yate's correction test. In all tests, a significance level of 0.05 was considered.

## 3. Results

### 3.1. High Levels of Expression and Purification of DTL-4 from *Escherichia coli* Soluble Fraction

**Figure 4:**
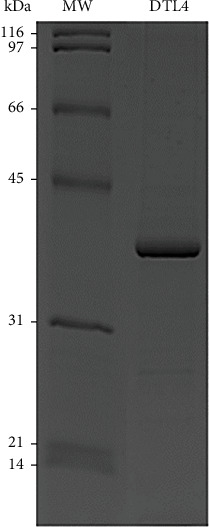
Dodecyl sulfate-polyacrylamide gel electrophoresis analysis of recombinant protein DTL-4. DTL-4 (~40 kDa) was purified in nickel affinity chromatography and submitted to SDS-PAGE analysis. Molecular weight markers are shown on the left.

The recombinant DTL-4 protein was expressed in *E. coli* BL21 (DE3) and purified from the soluble fraction on a Ni^2+^-charged Sepharose column using single-step chromatography. As shown in Figure 4, the protein migrated in SDS-PAGE as a single band with an apparent molecular weight of 40 kDa, as expected according to bioinformatic projections.

### 3.2. The DTL-4 Protein-Based ELISA Has High Accuracy for the Diagnosis of Human VL

**Figure 5:**
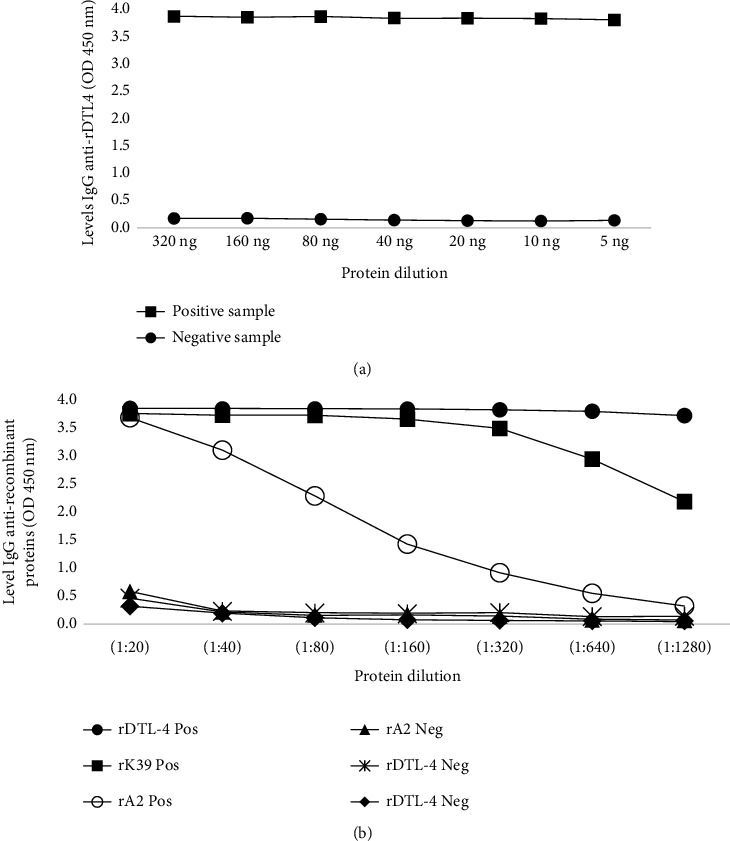
Titration curves for the determination of protein concentration and sera dilutions for DTL-4-ELISA. Pools of ten VL-positive and ten negative sera were assayed. (a) Titration of DTL-4 concentration by adding successive decreasing amounts of protein to wells on ELISA plates. (b) Titration of anti-DTL-4, anti-A2, and anti-K39 antibodies in pools of sera, using 10 ng of each protein per well.

The protein concentration to coat the ELISA plates was determined by a titration curve performed with pools containing ten positive and ten negative VL serum samples (Figure 5(a)). Moreover, since DTL-4 contains A2 epitopes and rK39 is the most widely used recombinant antigen for VL serodiagnosis, we have compared the antibody titers in the same sample pools against rA2, rK39, and DTL-4, using an ELISA made in-house. As shown in Figure 5(a), DTL-4 discriminates with high-precision positive and negative VL pools of sera, even when small amounts of protein were used to coat the ELISA plates. The DTL-4 concentration of 0.1 *μ*g/mL (10 ng/well) was then selected to coat the ELISA plates for further analyses.

A serum titration curve was also performed with the same pools of sera (Figure 5(b)). As seen in Figure 5(b), positive sera's reactivity with DTL-4 remains high, even at 1 : 1280 sera dilutions, while antibody titers against rA2 or rK39 dropped significantly after 1 : 80 or 1 : 320, respectively. The serum dilution of 1 : 100 was selected for the DTL-4-ELISA.

### 3.3. The DTL-4 Protein-Based ELISA Is Highly Sensitive for Serological Diagnosis of VL and Did Not Present Cross-Reactivity with Antibodies Present in Sera of Patients with Other Diseases

**Figure 6:**
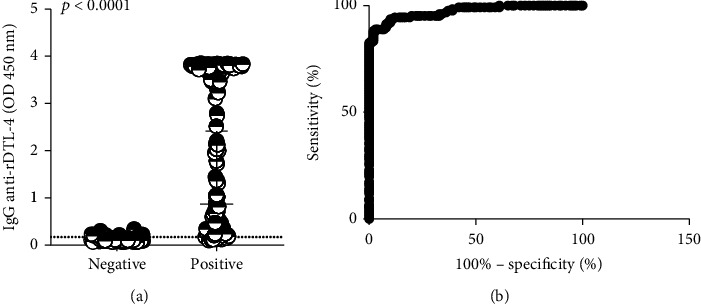
ELISA was performed to detect anti-DTL-4 IgG antibodies of sera. Sera from patients previously diagnosed with visceral leishmaniasis and healthy donors (panel 1.1). (a) DTL-4 discriminates significantly negative and positive samples (*p* < 0.0001; Mann–Whitney test). (b) ROC curve constructed from the absorbance values calculated for the 126 samples from patients with confirmed VL and 100 samples from healthy controls from the endemic area. Cut − off = 0.173. Area under the curve = 0.9727.

After determining the protein concentration and sera dilution to be used for best performance, DTL-4-ELISA was tested against 228 individual samples (128 positive and 100 negative) from sera panel 1.1 (Figure 1). In this assay, eleven samples out of the 128 positive ones did not react with DTL-4, corresponding to a sensitivity of 91.27% (95% CI: 84.92–95.56%), while eight samples among the negative ones reacted with DTL-4, leading to a specificity value of 92.00% (95% CI: 84.84%–96.48%) (Figure 6). This result indicated that DTL-4 efficiently discriminates negative and from positive samples (*p* < 0.0001; Mann–Whitney test).

Because it has been described that sensitivity may vary depending on the test used for the previous diagnosis and origin of the samples assayed, we tested the DTL-4-ELISA considering these parameters. Accordingly, when using ELISA rK39 as reference (51 samples from the State of Rio Grande do Norte, an endemic area for VL), the sensitivity was 93.90%.When the reference diagnostic test was the IT-LEISH® rapid test (150 samples from the State of Tocantins, endemic for VL), the sensitivity was 90.00%. When using the reference ELISA with *L. infantum* total extract (272 samples from the State of Tocantins, endemic for VL), the sensitivity was 83.30% (Table 2).

**Figure 7:**
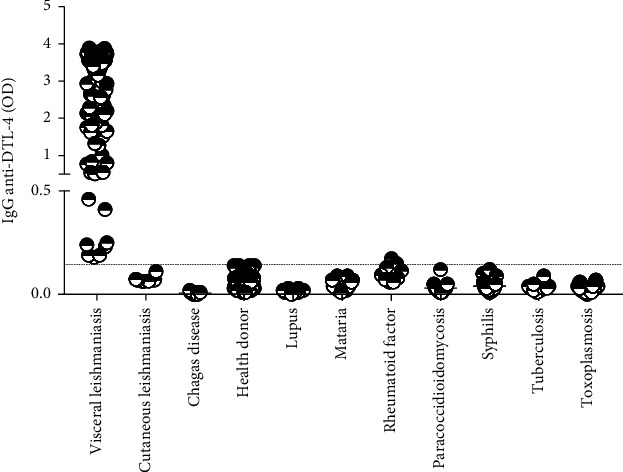
Cross-reactivity of DTL-4-ELISA with sera from patients diagnosed with other diseases. Sera from patients previously diagnosed with tegumentary leishmaniasis, Chagas' disease, malaria, toxoplasmosis, rheumatoid factor, lupus, tuberculosis, syphilis, and paracoccidioidomycosis were submitted for DTL-4-ELISA assay. Sera from VL patients (*n* = 155) and healthy donors (*n* = 140) were also included in this assay. The dotted line represents the cut-off.

Several conditions may lead to antibodies that cross-react in serological tests for VL, including other parasitic infections, whose transmission areas overlap with VL areas in Brazil. Thus, we tested the DTL-4 protein-based ELISA for specificity using sera of patients diagnosed with inflammatory disorders (lupus and rheumatoid factor) and other infectious diseases. As shown in Figure 7, there is no significant reactivity of the DTL-4 protein-based DTL-4-ELISA with sera of the selected inflammatory diseases. No significant reactivity was also detected by testing sera of patients diagnosed with syphilis, tuberculosis, paracoccidioidomycosis, or other parasitic diseases, including tegumentary leishmaniasis, Chagas' disease, malaria, and toxoplasmosis (panel 2.2, Figure 2), which are also highly prevalent in Brazil.

### 3.4. The DTL-4 Protein-Based ELISA Fulfills the Requirements for a Prototype of a Commercial Kit

To test the stability, repeatability, reproducibility, and homogeneity of DTL-4-ELISA, we used the biological samples from panel 1, described in Figure 1. After performing eight readings of two samples under the same conditions, they presented an average intra-assay coefficient of variation (CV %) of 3.8% and 4.6%, with the positive and negative samples, respectively. The test's reproducibility, evaluated by assaying three samples (one positive and two negative) for three consecutive days, presented a CV% of 0.6% with the positive sample and 7.1% and 8.0% with the negative ones. ELISA stability was assessed by accelerated aging at 37°C for 28 days, which corresponds to stability for 18 months if maintained under refrigeration (4 to 8°C).

### 3.5. External Validation of the DTL-4-ELISA for the Diagnosis of Human Visceral Leishmaniasis

To further validate the sensitivity and specificity of the DTL-4-ELISA prototype, additional sera, including a very well-characterized sera panel (panel 2, Figure 2), were used, including the samples with previous results of DAT and parasitological tests and samples from different geographical localities in Brazil. As shown in Table 3, high overall values for sensitivity (94.61%; 95% CI:89.94%-97.38%), specificity (99.41%; 95% CI: 96.39%-99.99%), and accuracy (97.02%; 95% CI: 94.61%-98.38%) were obtained.

**Figure 8:**
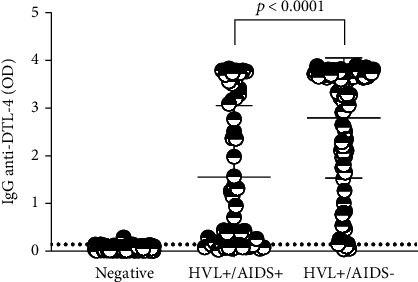
Evaluation of the reactivity of samples from coinfected patients from different locations assayed with DTL-4-ELISA. Sera from HVL+/AIDS- patients (*n* = 167), sera from HVL+/AIDS+ patients (*n* = 62), and sera from healthy donors (*n* = 169) were included in this assay. Sera samples were diluted at 1 : 100. The dotted line represents the cut-off.

We also compared anti-DTL-4 IgG in sera from patients with VL with those from patients coinfected with VL/AIDS (samples from panel 2.1, Figure 2). The evaluation of IgG anti-DTL-4 showed that the median absorbance of sera of patients previously diagnosed with VL/AIDS coinfection (median = 1.050) is lower than that of VL patients not coinfected (median = 3.430) (Mann–Whitney test, *p* < 0.0001) (Figure 8). The sensitivity of DTL-4-ELISA in 62 VL+/AIDS+ patients was 77.42% (95% CI: 65.48%-86.16%), while in 167 patients with VL, as detected by the parasitological method, but without HIV (VL+/AIDS), the sensitivity was 94.61% (95% CI: 89.94%-97.28%) (Fisher's exact test, *p* < 0.0001). Considering the results obtained in healthy donors, the accuracy of the DTL-4-ELISA in 62 VL+/AIDS+ patients was 93.51% (89.49%-96.10%).

We also evaluated the performance of the DTL-4-ELISA detection of VL in sera samples of VL and VL/AIDS patients from different localities in Brazil, according to the patients' origin (Table 4). DTL-4-ELISA displayed high sensitivity to detect VL infection in patients either from Campo Grande (97.26%) or Bauru (100.00%) localities. Overall sensitivity corresponded to 97.65% (*n* = 85). The test was also able to discriminate a significant proportion of VL/AIDS coinfected patients, either from Campo Grande (81.82%) or Bauru (100.00%). Considering both localities, overall sensitivity among VL/AIDS coinfected patients was 83.33% (*n* = 48). However, it should be noted that the number of VL/AIDS coinfected patients' sera from Bauru City was low, which may have impacted the sensitivity values observed.

### 3.6. Evaluation of the Performance of DTL-4 on Immunochromatographic Tests (ICT) for Human VL

**Figure 9:**
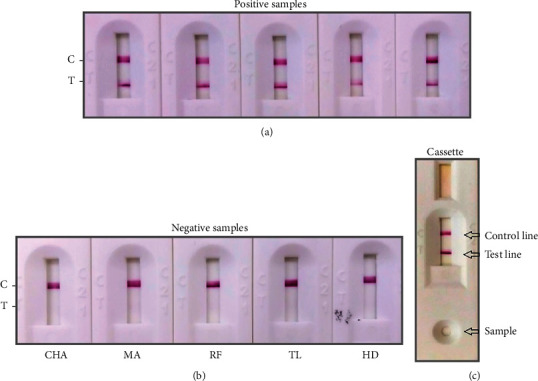
Illustrative results of detecting anti-*Leishmania* antibodies in sera and blood for VL diagnosis using the DTL-4-based ICT. (a) Sera samples from five VL different patients were included in this assay as a positive control (visible test and control line). (b) Sera samples from negative donors for LV with a previous diagnosis (ten samples of each disease) of Chagas' disease (CHA), malaria (MA), rheumatoid factor (RF), tegumentary leishmaniasis (TL), and healthy donor (HD) were tested. These assays presented negative results (only the control line was visible). (c) Representative test on the cassette. The sera sample from the VL patient was applied, and the control and test lines were visible.

Based on the performance of the DTL-4-ELISA developed, we tested the DTL-4 protein on an ICT test to detect antibodies in serum, peripheral blood, or plasma samples (Figures 9(a) and 9(c)). The ideal sample volume was 5 *μ*L of serum or plasma and 10 *μ*L of blood, both with reading times between 10 and 15 minutes. The ICT's test stability was assessed by accelerated aging at 37°C for 28 days, corresponding to stability for 18 months, under adequate storage conditions.

As an initial evaluation, the ICT was tested against sera from panel 1 (Figure 1). The results were considered positive only if the test and control lines' reactivities were observed and negative only if the control line's reactivity was observed, indicating the absence of specific antibodies (Figure 9(a)). The sensitivity, specificity, and accuracy values obtained with sera panel 1 were 91.95% (84.12 - 96.70), 97.65% (91.76 - 99.71), and 94.80% (95% CI: 90.21 - 99.65), respectively (Table 5).

Then, the ICT was tested against sera samples from validation panel 2 (Figure 2). The sensitivity, specificity, and accuracy values obtained with this panel were 91.98 (95% CI: 86.65 - 95.39%), 100% (95% CI: 96.30 - 100.00), and 95.14% (95%CI: 91.62 - 97.15), respectively (Table 5).

The results of DTL-4-ICT were then compared with the results of DTL-4-ELISA. Among the 154 samples positive by ELISA (HVL), nine samples were negative by DTL-4-ICT, while all the 100 samples negative by DTL-4-ELISA were also negative by DTL-4-ICT. Thus, the tests presented excelent agreement, with *k* = 0.898. The McNemar test results indicated no statistically significant difference between the methods (*p* = 0.2673).

On the other hand, when samples from coinfected VL/AIDS patients (all positive in VL-ELISA) were tested (*n* = 46), the sensitivity of DTL-4-ICT was lower (73.91%) as compared to DTL-4-ELISA 77.42%. A good concordance of *k* = 0.657 was observed between ELISA and ICT sensitivity (Table 5).

The evaluation of the interference of antibodies against other diseases (Chagas' disease, tegumentary leishmaniasis, malaria, and rheumatoid factor) that potentially would cross-react with DTL-4-ICT showed no cross-reactivity with antibodies raised against these other diseases (Figure 9). When sera samples of VL patients spiked with peripheral blood were tested (*n* = 67), the sensitivity determined for the ICT was 90% and the specificity was 100%. An illustration of the reactivity of a sera sample spiked with blood is seen in Figure 9(c).

## 4. Discussion

This study is aimed at evaluating a new chimeric recombinant protein, named DTL-4, as a potential antigen for accurate serological diagnosis of VL. DTL-4 contains epitopes from A2 and other proteins expressed by visceralizing species of *Leishmania*, which have been identified in previous immunoproteomic studies [21, 32, 33]. A2 has been consistently shown to discriminate symptomatic and asymptomatic VL individuals, either among human or dog populations [9, 16, 21, 34]. As shown for other antigens such as the *Leishmania* kinesins, the repetitive amino acid structure may amplify antibody detection, improving diagnosis sensitivity [22, 35]. On the other hand, the fact that A2 is present only in visceralizing *Leishmania* species also improves diagnosis specificity [23]. Besides its antigenic properties, DTL-4 is a recombinant antigen that is easily purified as a soluble protein and displays a low isoelectric point and significant amounts of positive and negative amino acids, which are highly desirable aspects for antibody reactivity and scaling up production, enabling cheaper and faster production in industrial settings and its further assembling of diagnostic tests in different formats.

By using well-characterized sera panels, the DTL-4-based ELISA specifically discriminated negative from positive samples in a ratio of 25 and displayed an improved performance (sensitivity, specificity, and accuracy of 94.61%, 99.41%, and 97.02%, respectively), when compared to other commercial ELISA kits for VL serodiagnosis available in Brazil, as reported by Freire et al. [36]. DTL-4 also performed better than rK39-based ELISA [37] in samples from Minas Gerais and Mato Grosso states, with 90.67% accuracy (*p* = 0.0033).

In general, ICT is a test format easier to perform than ELISA or other diagnosis platforms and can be applied individually, at the bedside, and in outpatient clinics. ICT has also been proven to be a versatile test format for use in field conditions. Although ICT is regarded as a more suitable format for serological screening, high sensitivity and specificity can be achievable and, in some cases, may replace more complex diagnostic tests. Boelaert et al. [38] claims that an ideal VL diagnostic test should perform with a sensitivity ≥ 95% and a specificity ≥ 98%. Compared with these parameters, the DTL-4 antigen displayed a more than satisfactory performance in both test formats, ELISA and ICT. It is noteworthy that a minimum accuracy of 90% is required for ICT tests to be selected for the Brazilian Government's purchase through public calls to supply the public health system with commercial tests for VL diagnosis [39]. In 2019, Freire et al. [36] reported an accuracy of 96.2% (92.0–98.3) using IT-LEISH and 93.7% (88.8–96.6) with Kalazar Detect. Therefore, it may be considered that DTL-4-ICT displayed excellent performance, with an accuracy of 95.14%.

The majority of the ICTs available worldwide for active VL serodiagnosis, including the Kalazar Detect (InBios International, Seattle, WA) [23], IT-LEISH (Bio-Rad Laboratories, Inc.) [40], and Onsite *Leishmania* IgG/IgM Combo Rapid Test (CTK Biotech) [39] are based mainly on the same kinesin antigens also used in available ELISA. In a recent and comprehensive study, WHO reported that five different RDTs based on rK39 or rKE performed with high specificity (>95%) in all the different regions of the globe tested; however, sensitivity varied between tests (range, 36.8%-100%) and between regions. In ISC, all tests had a high sensitivity (>93%); in East Africa and Brazil, sensitivity results were variable, but no test exceeded 92% sensitivity (95% CI: 87.8-94.8%) [20].

The Brazilian Ministry of Health currently provides the OnSite *Leishmania* IgG/Igm Combo Rapid Test kit to diagnose suspected VL patients attending the public health system [41]. When evaluated in a panel of sera samples of VL cases from different Brazil regions, this test resulted in 92.1% sensitivity and 95.9% specificity, considering the IgG band. However, no VL case was positive on the IgM test, and one patient diagnosed with *Mycobacterium* infection was IgM positive [39], disclosing, therefore, the sensitivity and specificity of anti-*Leishmania* IgM antibodies for VL diagnosis. In 2019, Porcino et al. [42] also evaluated the ICT Kalazar Detect and Onsite *Leishmania* IgG/IgM Combo Rapid Test performance and obtained 76% and 64% sensitivities, respectively, in patients with active VL.

Differences were observed for the sensitivity of the DTL-4-based tests when sera samples from patients living in different regions in Brazil were assayed. This finding may be attributed to the small sample size of some subset panels used. However, this result corroborates previous reports showing variations in sensitivity and specificity when similar antigens are tested with sera panels from people living in different regions [20, 43, 44]. These discrepant patterns may be attributed to differences in *Leishmania* strains causing infection or other epidemiological factors not very well understood, such as heterogeneity of populations, MHC molecules, age, and nutritional status. Nonetheless, additional epitopes present in chimeric recombinant proteins, such as DTL-4, may improve serodiagnosis performance.

The interference analysis performed to evaluate potential cross-reactivity showed that DTL-4, regardless of ICT and ELISA tests, very well discriminated VL from other diseases, including Chagas' disease, malaria, and tegumentary leishmaniasis, which are parasitic diseases also quite prevalent in different regions of Brazil. This finding indicated that DTL-4-ICT performed similarly to Kalazar Detect when tested with samples from the same diseases, as no cross-reactivity was observed in both studies [23]. Also, in samples from Chagas disease, DTL-4-ICT was comparable to IT-LEISH [45]. On the other hand, Pedras et al. [37] reported lower specificity with malaria (85.0%) and Chagas' disease (83.3%) samples by using an rK39-based ELISA.

AIDS coinfection may impair the production of anti-*Leishmania* antibodies in patients with VL. Thus, a worrying aspect that still poses a significant challenge is the low sensitivity of serological tests in VL/AIDS coinfected patients [46]. In Brazil, the prevalence of VL/AIDS is 9% [47]. However, the percentage of coinfection is underestimated because it considers only patients with VL manifestations. Also, around 40% of VL patients are negative by HIV serology, and both *Leishmania* and HIV infection may be asymptomatic [47]. Patients with negative results should undergo further investigation, such as direct detection of parasites in bone marrow aspirates. However, a positive result combined with clinical signs has a significant diagnostic value [48].

In this study, DTL-4-ELISA presented an overall sensitivity of 77.42% and DTL-4-ICT presented an overall sensitivity of 73.91% for diagnosing VL patients with HIV. Although sensitivity was lower in the group of coinfected patients compared to that observed for the non-co-infected VL patients, it seems that DTL-4 may slightly improve VL serodiagnosis in this patient group, as compared to other tests, including the commercially available kits. Freire et al. [36] reported the sensitivity values of 65.8% for NovaLisa *Leishmania infantum* IgG (ELISA) in HIV-coinfected patients from Brazil. These authors also reported that low sensitivity values were obtained with IT-LEISH (63.2%) and Kalazar Detect (47.4%). One possible alternative to improve the DTL-4-based test performance, which was not thoroughly investigated in the present work, would be to set up different conditions for DTL-4-based tests for samples from patients suspected of HVL/AIDS coinfection. Alterations may include changes in protein amounts, sera dilutions, or sample volume, which may be more sensitive for detecting anti-DTL-4 antibodies in HIV-coinfected patients. Additional studies may be performed to evaluate the use of DTL-4-based tests in epidemiological surveys, detect asymptomatic infection, and monitor *Leishmania* transmission through blood transfusion or treatment response.

In summary, the prototyped DTL-4-based ELISA or ICT displayed high accuracy and excellent performance compared to some in house or commercially available *Leishmania*-derived recombinant antigen-based ELISA or ICT tests. Although there is still much space for additional technical and product improvements, the DTL-4-based tests described herein represent improved alternative options for precise VL serological diagnosis.

## Figures and Tables

**Table 1:** Physicochemical evaluation of the DTL-4 protein.

Parameters	Results
Number of amino acids	403
Estimated molecular weight (kDa)	40501.09
Theoretical isoelectric point	4.85

**Table 2:** Performance of DTL-4-ELISA in the diagnosis of VL according to the reference diagnostic test, like rK39-ELISA, IT-LEISH® rapid test, and *L. infantum*-ELISA (total extract).

	DTL-4-ELISA	
	Sensitivity% (*n*)	Specificity% (*n*)
rK39-ELISA	93.90 (51)	90.40 (100)
IT-LEISH® rapid test	90.00 (150)	94.00 (100)
*L. infantum*-ELISA	83.30 (272)	95.30 (100)

**Table 3:** Performance of the DTL-4-ELISA in samples from patients with visceral leishmaniasis and negative controls from different geographical regions in Brazil.

Locality	Sensitivity% (*n*)	95% CI	Specificity% (*n*)	95% CI
Aracaju, SE	100.00 (9)	65.54-100.00	91.67 (12)	62.47-100.00
Campo Grande, MS	97.26 (73)	89.98-99.82	100.00 (121)	96.30-100.00
Bauru, SP	100.00 (12)	71.80-100.00	100.00 (7)	59.56-100.00
Natal, RN	93.75 (16)	69.69-99.99	100.00 (29)	88.13-100.00
Piauí state	89.47 (57)	78.53-95.44	—	—
Total	94.61 (167)	89.94-97.28	99.41 (169)	96.39-99.99

SE: Sergipe state; MS: Mato Grosso do Sul state; SP: São Paulo state; RN: Rio Grande do Norte state; *n*: number of samples; 95% CI: 95% confidence interval.

**Table 4:** Performance of DTL-4-ELISA to diagnosis of VL in samples of AIDS patients from different locations in Brazil.

Location	Subjects	Diagnostic accuracy (95% CI)		
		Sensitivity% (*n*) 95% CI	Specificity% (*n*) 95% CI	Accuracy% 95% CI
Campo Grande (Mato Grosso do Sul state)	VL	97.26 (73) 89.98-99.82	—	98.84 95.61-99.95
	VL/AIDS	81.82 (44) 67.78-90.75	—	94.44 89.26-97.32
	Healthy		100.00 (121) 96.30-100.00	—

Bauru (São Paulo state)	VL	100.00 (12) 71.80-100.00	—	100.00 80.21-100.00
	VL/AIDS	100.00 (04) 45.41-100.00	—	100.00 69.98-100.00
	Healthy		100.00 (07) 59.56-100.00	—

Total	VL	97.65 (85) 91.32-99.86	—	99.06 96.42-99.97
	VL/AIDS	83.33 (48) 70.16-91.57	—	95.48 91.20-97.83
	Healthy		100.00 (128) 96.50-100.00	—

VL: visceral leishmaniasis; VL/AIDS: coinfection; healthy donors; *n*: number of samples; 95% CI: 95% confidence interval.

**Table 5:** Sensitivity, specificity, accuracy, and agreement of the VL-ICT versus the VL-ELISA using human samples from *L*. *infantum*-infected patients and healthy donors and VL/AIDS+), from panels 1 and 2.

VL (*n* = 87) and healthy donors (*n* = 85) (panel 1)		

Assay type	ELISA (+)	ELISA (-)
ICT (+)	80	02
ICT (-)	07	83
Accuracy = 94.80% (95% CI: 90.21–99.65%)	Sensitivity = 91.95% (95% CI: 84.12–96.70%)	Specificity = 97.65% (95% CI: 91.76–99.71%)

VL (*n* = 162) and healthy donors (*n* = 100) (panel 2)		

Assay type	ELISA (+)	ELISA (-)
ICT (+)	145	4
ICT (-)	9	104
Total	154	108
Accuracy = 95.14% (95% CI: 91.62–97.15%)	Sensitivity = 91.98% (95% CI: 86.65–95.39%)	Specificity = 100.00% (95% CI: 96.30–100.00%)

VL/AIDS+ (*n* = 46) and healthy donors (*n* = 64) (panel 2)		

Assay type	ELISA (+)	ELISA (-)
ICT (+)	34	6
ICT (-)	12	58
Total	46	64

Accuracy = 83.64% (IC 95%: 75.52–89.48%)	Sensitivity = 73.91% (IC 95%: 59.74–84.40%)	Specificity = 90.63% (IC 95%: 81.02–95.63%)

Concordance	Kappa index-*k* (95% CI)	Value *p*^∗^
ELISA versus ICT (VL)	0.898 (0.844–0.952)	0.2673
ELISA versus ICT (VL/AIDS+)	0.657 (0.514–0.801)	0.2386

VL: visceral leishmaniasis; ELISA: enzyme-linked immunosorbent assay; ICT: immunochromatographic test; All the 46 VL/AIDS+ samples were positive by VL-ELISA. ^∗^McNemar test was used to estimate the statistical differences between ELISA and ICT. The differences were considered statistically significant when value *p* < 0.05.

## Data Availability

The data used to support the findings of this study are available from the corresponding author upon request.
